# Encystment and Excystment Processes in *Acanthamoeba castellanii*: An Emphasis on Cellulose Involvement

**DOI:** 10.3390/pathogens14030268

**Published:** 2025-03-10

**Authors:** Mathew Choaji, Ascel Samba-Louaka, Zineb Fechtali-Moute, Willy Aucher, Sébastien Pomel

**Affiliations:** 1 Université Paris-Saclay, CNRS BioCIS, 91400 Orsay, France; 2 Université de Poitiers, UMR CNRS 7267, Laboratoire Ecologie et Biologie des Interactions, 86000 Poitiers, France

**Keywords:** *Acanthamoeba castellanii*, cyst wall, cellulose, encystment, excystment

## Abstract

The free-living amoeba *Acanthamoeba castellanii* is a unicellular eukaryote distributed in a wide range of soil or aquatic environments, either natural or human-made, such as rivers, lakes, drinking water, or swimming pools. Besides its capacity to transport potential pathogens, such as bacteria or viruses, *Acanthamoeba* spp. can have intrinsic pathogenic properties by causing severe infections at the ocular and cerebral level, named granulomatous amoebic encephalitis and amoebic keratitis, respectively. During its life cycle, *A. castellanii* alternates between a vegetative and mobile form, named the trophozoite, and a resistant, latent, and non-mobile form, named the cyst. The cyst wall of *Acanthamoeba* is double-layered, with an inner endocyst and an outer ectocyst, and is mainly composed of cellulose and proteins. The resistance of cysts to many environmental stresses and disinfection treatments has been assigned to the presence of cellulose. The current review aims to present the importance of this glycopolymer in *Acanthamoeba* cysts and to further report the pathways involved in encystment and excystment.

## 1. Introduction

Free-living amoebae (FLA) are unicellular microorganisms that can live autonomously in the environment, as opposed to parasitic amoebae that require a host to survive [1]. They are ubiquitous in various aquatic and terrestrial environments, exhibiting a range of ecological roles [2,3]. FLA primarily feed on microorganisms such as bacteria through phagocytosis [4,5]. However, some pathogenic bacteria including *Legionella pneumophila*, *Pseudomonas aeruginosa*, and *Mycobacterium* spp. can resist phagocytosis and multiply in FLA [5,6]. This highlights the role of FLA as potential carriers of pathogenic bacteria in natural environments [5,7], where bacteria can evade predation and survive adverse conditions, thereby increasing their persistence and potential for transmission and raising concerns about public health and water quality management [8,9]. FLA are commonly found in water sources, and their association with pathogenic bacteria suggests a potential risk for waterborne infections [10,11]. Several works reported that intracellular growth in the FLA *Acanthamoeba castellanii* influences the entry mechanism and enhances the virulence of *L. pneumophila* [12,13,14]. These findings underscore the importance of understanding the interactions between microorganisms in complex ecosystems. The ability of pathogenic bacteria to exploit FLA for survival and replication demonstrates the interconnectedness of microbial communities and the potential for cross-species interactions to influence disease dynamics.

Besides their role as potential reservoirs of pathogenic microorganisms, some FLA species stand out as pathogens capable of causing severe human infections, such as granulomatous amoebic encephalitis (GAE) caused by *Acanthamoeba* spp. and *Balamuthia mandrillaris* and primary amoebic meningoencephalitis (PAM) caused by *Naegleria fowleri. Acanthamoeba* spp. can also cause amoebic keratitis (AK), a serious ocular infection associated with corneal trauma, frequently associated with improper use of contact lenses [15,16,17]. For cerebral infections, FLA enter the body either through skin lesions (*Acanthamoeba* spp. or *B. mandrillaris*) or after inhalation of contaminated water or soil and will further colonize the upper (PAM) or lower (GAE) respiratory tract before reaching the brain [16]. While only several hundreds of FLA cerebral infections have been reported so far worldwide, the annual incidence of AK is currently estimated at more than 20,000 cases worldwide, corresponding approximately to a prevalence of 2.9 cases per million individuals, with notable variabilities between countries, and to 2% of all corneal infections [18,19,20]. In the 1990s, the AK incidence was estimated at only one or two cases per million lens wearers. This increase was mainly ascribed to higher popularity of contact lens wearing, but also to a greater awareness and better diagnosis techniques. Combination therapies, empirically associating a large diversity of drugs, are commonly used for cerebral FLA infections [18]. Likewise, combinations of drugs like chlorhexidine with polyhexamethylene biguanide (PHMB) or propamidine have been commonly used to treat amoebic keratitis [21,22]. However, treating amoebic keratitis requires frequent and prolonged drug administration, with infection relapse in 10% of the cases [23]. Nonetheless, a high dose of PHMB (0.08%) monotherapy has recently shown very promising results in clinical practice and has received a marketing authorization for amoebic keratitis treatment by the European Commission [24,25].

Various strategies, including chlorine, heat shock, and peracetic acid with hydrogen peroxide, are employed to disinfect FLA in warm water networks [26]. Nevertheless, resistance to these disinfectants is emerging as a challenge [27]. These difficulties stem from the free-living amoebae’s ability to undergo encystment, a pivotal survival strategy in response to adverse environmental conditions such as nutrient depletion, osmotic stress, and chemical stressors including bacterial toxins [11]. This differentiation process complicates treatment efficacy and makes eradicating the amoebae difficult. Therefore, understanding the fundamental biology of FLA, particularly their life cycle dynamics, is crucial for the development of effective strategies to mitigate their pathogenic potential.

FLA exhibit a complex life cycle involving encystation, also known as encystment, where trophozoites convert into cysts allowing them to withstand unfavorable conditions, and excystation (or excystment), where cysts convert back into trophozoites, enabling amoebae to resume their active, vegetative state when environmental conditions improve [28,29,30,31].

**Figure 1:**
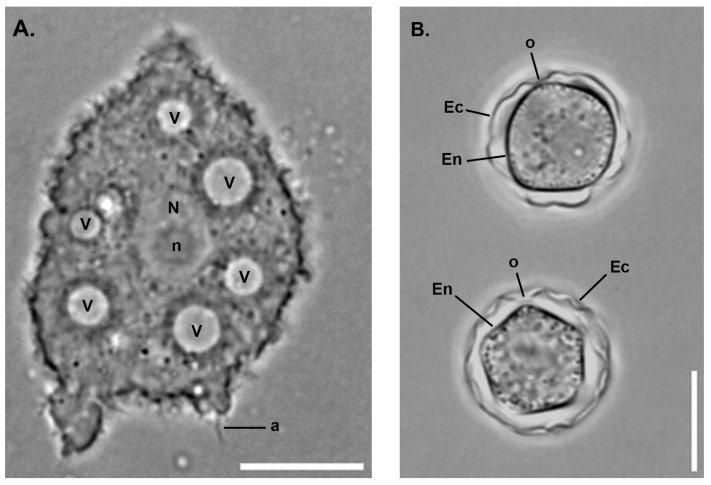
**Phase contrast images of *Acanthamoeba castellanii*.** (**A**) Trophozoite presenting acanthopodia (a) allowing mobility, with several vacuoles (V) around the nucleus (N) presenting a nucleolus (n) in its center. (**B**) Two cysts characterized by a double layer with the outermost ectocyst (Ec) and the innermost polygonal endocyst (En). These two layers are connected at the ostiole (o). Scale bar = 10 µm.

Within FLA’s life cycle, the trophozoite stage corresponds to a vegetative form capable of feeding, multiplying, and moving by amoeboid movement (Figure 1A). Trophozoites are particularly important in the pathogenesis of diseases caused by these protozoa, as they are often responsible for tissue damage and clinical symptoms and the internalization and transport of potential pathogenic microorganisms [32]. On the other hand, the cyst stage is a dormant and resistant form that enables the protozoa to survive harsh environmental conditions (Figure 1B). Remarkably, cysts can remain viable in the environment for up to 20 years while maintaining their pathogenicity [33]. Additionally, some FLA from the family Vahlkampfiidae group, such as *N. fowleri*, can produce flagellated forms, which further enhance their ability to disseminate to various environmental conditions [34].

While trophozoites are sensitive to disinfection or treatment, cysts are resistant to diverse disinfection or treatment conditions [23,27]. Indeed, cysts of the FLA from the genus *Acanthamoeba* have been shown to be especially resistant to several environmental stresses or disinfection methods, more than other FLA such as *N. fowleri* or *Vermamoeba vermiformis* [27,35]. Aksozek et al. (2002) investigated the resistance of *A. castellanii* cysts to various physical, chemical, and radiological conditions, revealing their remarkable resilience to environmental stresses [36]. The study shows that *Acanthamoeba* cysts can endure prolonged desiccation without losing viability and tolerate a wide range of temperatures. Additionally, cysts exhibit resistance to many chemical agents and UV radiation [36], allowing them to persist in environments subjected to water treatment and disinfection protocols in healthcare settings. Indeed, FLA have been identified at different steps of drinking water distribution systems, from treatment plants to tap water [37,38], and an enrichment of FLA has been especially observed in biofilms developed at upper levels of drinking water storage towers [39]. This unequal distribution would be due to variations of water level in the storage tower leading to organic matter deposition of the support, and also, for some FLA genera such as *Acanthamoeba*, a decrease in cyst density as a function of their age [40]. These findings underscore the robustness of *Acanthamoeba* cysts and the need for enhanced water treatment strategies, stricter monitoring to ensure the safety of drinking water and other public water systems, and the development of more effective sterilization methods to prevent nosocomial infections.

The resistance of cysts has been attributed to the presence of cellulose in the cyst wall, as an increase in cellulose content coincides with biocide resistance [17,35,41,42]. In *A. castellanii*, cellulose is reported to make up to 35% of the cyst wall, and variations in the cellulose composition of cyst walls result in differing resistance to biocides among closely related *Acanthamoeba* species [42]. One study identified that resistance to various biocides and moist heat was developed between 12 h and 36 h, corresponding with increased levels of cellulose in the cell samples at approximately 14 h to 16 h [17]. These data are in line with several works showing an upregulation of transcripts related to carbohydrate biosynthetic pathways within the first hours of encystment [43]. Moreover, a functional analysis using siRNA showed that both cellulose synthase and xylose isomerase activities are crucial for *A. castellanii* encystment [44]. Furthermore, a digestion of *A. castellanii* cysts with low concentrations of cellulase (7.5 U/mL) stimulated their excystment [10], reinforcing the hypothesis that cellulose is a main component of *A. castellanii* cyst walls. Indeed, cellulose synthesis has been shown to be essential for the differentiation of viable cysts in other amoebae [45]. While the genome of *A. castellanii* encodes for cellulose-synthase and cellulase, it is also composed of genes involved in the synthesis of another glycopolymer involved in cyst wall composition of several protozoa cysts [46,47,48]: chitin. As both cellulase and chitinase activities have been measured during *A. castellanii* excystment [29], and as the chitin-binding WGA (wheat germ agglutinin) lectin can bind to their cyst wall [49,50], the presence of chitin in *Acanthamoeba* cyst walls cannot be excluded. However, an exogenous treatment with 0.1 U/mL to 2 U/mL of chitinase did not have any effect on *A. castellanii* cysts [10]. Although the chitinase concentrations used in this work were low, the proportion of chitin in *A. castellanii* cyst wall remains to be clarified and its role during encystment or excystment is currently under debate.

Understanding the mechanisms underlying cyst resistance is important for developing effective strategies to control *Acanthamoeba* infections and mitigate their impact on human health [51]. This review focuses on both the encystment and the excystment processes of *A. castellanii*, exploring the findings from scanning electron microscope studies, morphological observations, molecular investigations, and potential therapeutic strategies targeting cellulose degradation and biosynthesis pathways.

## 2. Structure of the Cyst Wall

**Figure 2:**
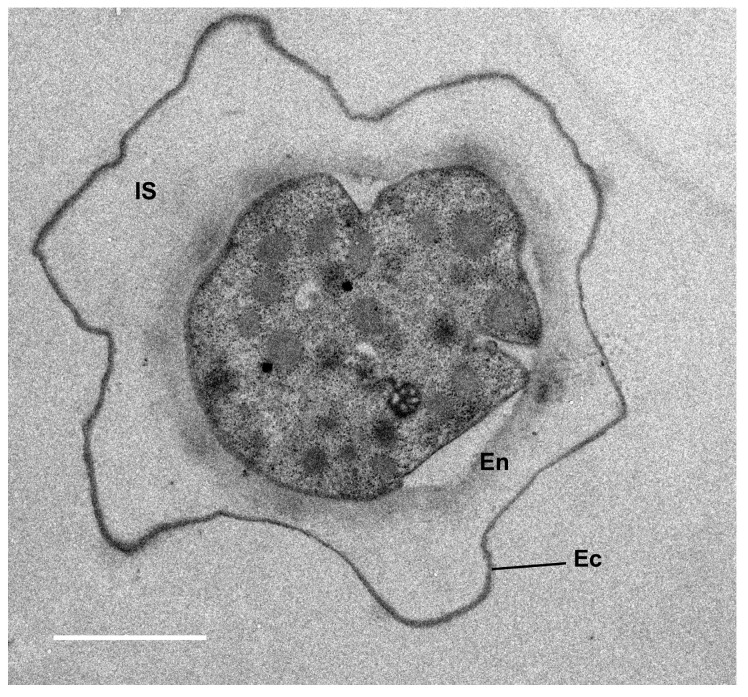
**Transmission electron microscopy image of a cyst of *Acanthamoeba castellanii*.** The cyst wall of *A. castellanii* is composed of two layers, the inner endocyst (En) and the outer ectocyst (Ec), separated by the intercystic space (IS). Scale bar = 2 µm. (Adapted from [10]).

**Figure 3:**
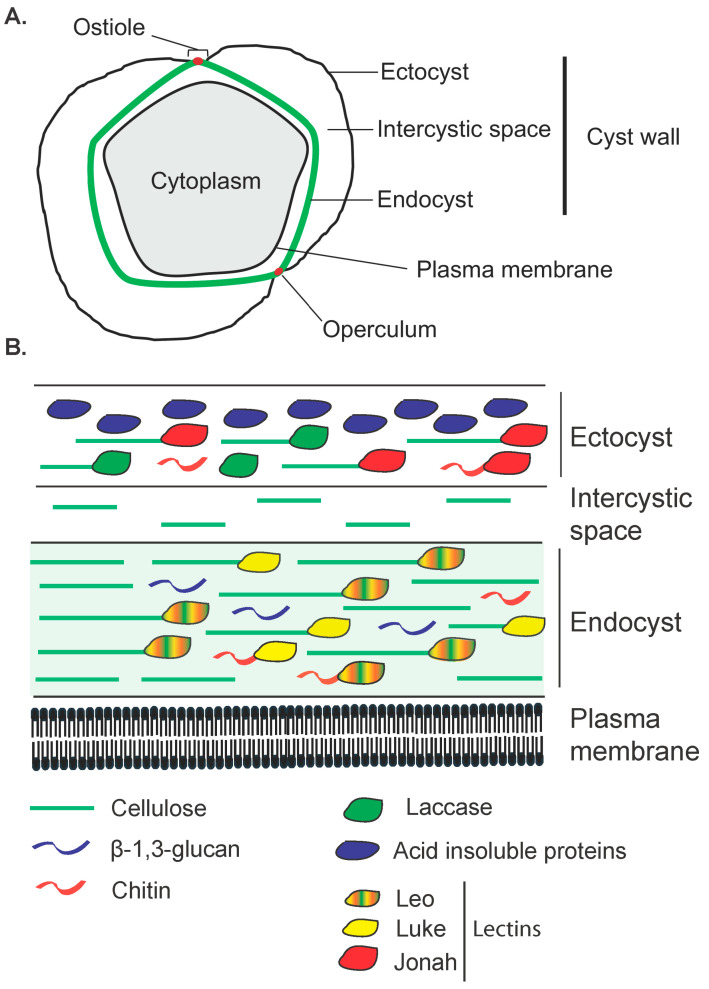
**Illustration depicting the different layers of the *Acanthamoeba castellanii* cell wall.** (**A**) *A. castellanii* cyst shows ectocyst and endocyst (in green) separated by an intercystic space. The two layers are closely apposed at the ostiole which is covered by a cap, named the operculum (in red). (**B**) The cyst wall contains structural sugar polymers (cellulose, glucans, putative chitin) associated with proteins such as lectins.

The cyst wall of *A. castellanii*, like many other amoebae, is composed of two layers, the ectocyst and the endocyst, which are separated by an intercystic space (Figure 2; Figure 3A) [11,52,53,54]. Ectocyst and endocyst layers merge at the level of ostioles, corresponding to pores used to monitor environmental conditions. Each ostiole is covered by a cap, named the operculum, which is removed during excystment to allow trophozoite emergence. In *Acanthamoeba*, the ectocyst is the outermost layer of the cyst wall composed of acid-insoluble proteins and polysaccharides, including cellulose, and serves as a protective layer (Figure 3B) [30]. The endocyst is the innermost layer, composed mainly of cellulose, and plays a critical role in maintaining the integrity of the cyst and protecting the enclosed trophozoite (Figure 3B). The intercystic space is filled with proteins, lipids, carbohydrates, and some traces of cellulose which provides additional support to the cyst. The endocyst is thicker and the ectocyst is thinner and wrinkled [55]. Although cellulose appears to be the main polysaccharide of the *Acanthamoeba* cyst wall, the presence of other carbohydrates cannot be excluded, as *Acanthamoeba* cysts can be labeled with probes binding to chitin (WGA) or both β-1,3- and β-1,4-glucans (Calcofluor white; Figure 3B) [49,56,57]. Moreover, three main lectins, named Jonah, Luke, and Leo, as well as an abundant copper oxidase, named laccase, have also been specifically localized to the endocyst and ostioles or to the ectocyst (Figure 3B) [49]. The morphology of the FLA cyst walls depends on their genus and species and has been used for their identification [11]. Observations under the light microscope revealed that in *Acanthamoeba* spp. cysts are spherical or ovoid with characteristic polygonal endocysts (Figure 1B), although they may sometimes be deformed [41].

## 3. Encystment

The three phases of encystment in *A. castellanii* are the induction phase, the wall-synthesis phase, and, ultimately, a dormant stage characterized by decreased metabolic activity. These phases occur in sequence, each stage being estimated to last approximately 4–6 h, 20–24 h, and 2–7 d, respectively [17].

**Figure 4:**
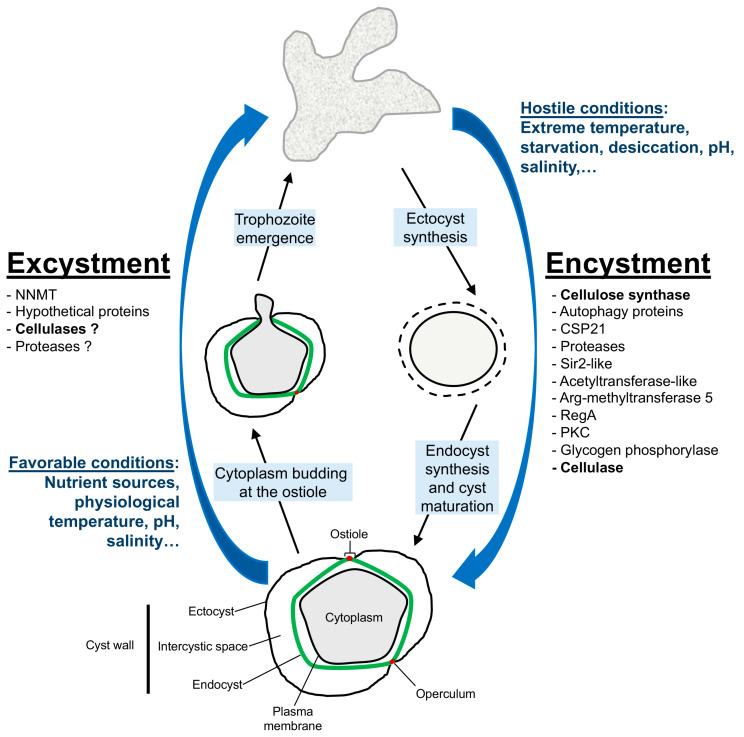
**Main cellular steps and endogenous proteins involved in the encystment and excystment processes in *Acanthamoeba castellanii*.** Encystment starts with the formation of the outer layer, the ectocyst, followed by the inner layer, the endocyst. Excystment is characterized by the emergence stage, when the operculum is removed and a cytoplasmic bud appears at the ostiole, and the post-emergence stage when the trophozoite is released and the remaining cyst wall disappears. The operculum and the endocyst are represented in red and green, respectively. NNMT = nicotinamide N-methyltransferase; CSP21 = Cyst-Specific Protein of 21 kDa; Sir2-like = Deacetylase; Arg-methyltransferase 5 = Arginine methyltransferase 5; RegA = AMP phosphodiesterase; PKC = Protein kinase C (from [10,43,44,54,60,61,62,63,64,65,66,67,68,69,70,71,72,73,74,75,76,77,78,79,85,86,87,88]).

Biochemical changes accompany the encystment process, involving intricate regulatory mechanisms and molecular pathways [43,58,59]. Indeed, *A. castellanii* rapidly responds to starvation, a nutritional stress-inducing encystment, through regulation of protein phosphorylation and gene expression at the transcriptional level [43]. Transcripts related to carbohydrate metabolic processes, presumably reflecting the formation of cellulose cell wall, were upregulated as early as 1 h after starvation [43]. Previous studies have also shed light on various aspects of encystment, highlighting the roles of many proteins such as autophagy proteins [60,61,62,63], ubiquitin-like proteins [64], or a specific cyst protein named CSP21 (Cyst-Specific Protein of 21 kDa) [65], or diverse enzymes with protease [66,67,68,69], deacetylase [70], acetyltransferase [71], methyltransferase [72], phosphodiesterase [73], kinase [74], or glycogen phosphorylase [75] activities (Figure 4). The latter has been found to be essential for glycogen breakdown to provide glucose for cellulose synthesis during *A. castellanii* encystment. Likewise, cellulose synthesis and cellulose synthase activity have also been found to increase during encystment and to be essential for this cellular process [44,76,77,78,79] (Figure 4).

Encystment is a tightly regulated process driven by various signaling pathways that lead to the synthesis and assembly of the cyst wall, a structure primarily composed of cellulose and other polysaccharides in *Acanthamoeba* [11,57,80]. This process begins with the formation of the outer layer, the ectocyst, followed the development of the inner layer, the endocyst, in mature cysts [54] (Figure 4). Studies by Chavez-Munguia et al. (2013) and Garajová et al. (2019) have highlighted the importance of cellulose fibrils in cyst wall architecture and the organization of the cytoskeleton during encystment [41,52]. Cellulose fibrils were detected in both cyst wall layers, namely the endocyst and the ectocyst, as well as in the intercystic space [41]. Moreover, three main lectins constituting the *Acanthamoeba* cyst wall, named Jonah, Luke, and Leo, and a laccase were described to bind to cellulose and, to a lesser extent, to chitin, and to be highly regulated during encystment (Figure 3B) [49]. Recent cellular studies have confirmed that ectocyst localization of Jonah and laccase is caused by early encystment-specific expression and localization to the endocyst and ostioles of Luke and Leo is caused by later expression [50]. Structural analyses of the lectins have identified the carbohydrate-binding modules of the lectins, named β-jelly-roll folds (BJRFs) in Luke, four disulfide knots (4DKs) in Leo, and a single β-helical fold (BHF) in Jonah, which bind to glycopolymers in both layers of the deproteinated cyst wall. The presence of aromatic amino acids in BJRFs and 4DKs is necessary for cellulose binding and proper localization of Luke and Leo in the cyst wall [50]. Moreover, evolutionary studies suggested a bacterial origin of Jonah BHFs, a common ancestry with slime molds for Luke BJRFs, while Leo 4DKs were unique to *Acanthamoeba* [50]. Interestingly, Deichmann and Jantzen (1977) discovered that cellulases, enzymes that degrade cellulose, are also expressed during encystment, which seems paradoxical [81]. This simultaneous expression of cellulose synthase and cellulases can probably be explained by their tightly regulated spatial and temporal activity, where controlled cellulose degradation could be necessary for cyst wall remodeling and maturation. Cellulases may also remove inadequately-structured cellulose, ensuring high-quality cyst wall formation. Furthermore, cellulases may have additional roles beyond cellulose degradation, and could be involved in signaling pathways, with potential non-cellulolytic roles during encystment. This complexity underscores the need for further research into these regulatory networks of *Acanthamoeba* encystment.

## 4. Excystment

Excystment is a poorly studied process that is triggered by environmental signals such as nutrient availability [30,82,83] or the presence of bacteria [84]. Chambers and Thompson have provided a detailed study of the excystment stages in *A. castellanii* using scanning electron microscopy and identified four distinct phases and specific structural features involved in this process [85]. In the mature cyst stage, cysts display multiple ostioles covered by an operculum, with a characteristic double-layered cyst wall comprising an inner endocyst and an outer ectocyst. During the pre-emergence stage, cysts are in the optimal growth medium, but do not show visible signs of trophozoite emergence. The emergence stage is marked by the appearance of a cytoplasmic bud at the ostiole, where the operculum has been removed, indicating the initiation of the amoeba’s emergence. Finally, in the post-emergence stage, free trophozoites are observed having exited the cysts and leaving behind empty cyst walls with irregularly shaped holes.

Several important observations were also noted in this study: first, trophozoites were observed emerging specifically from the ostioles, suggesting a regulated and presumably enzymatic process facilitating the removal of the operculum and subsequent emergence of the trophozoite. Second, similar to what was observed by Mattar and Byers [82], empty cyst walls remain visible for several hours after excystment initiation. Third, not all cysts excyst synchronously, suggesting variability in the timing of excystment. This could be due to differences in individual cysts’ readiness or environmental conditions.

Using mRNA sequencing, five genes were recently shown to be upregulated during excystment [86]. The silencing of these genes, by using specific siRNA, revealed an essential role for four of them (ACA1_031140, ACA1_032330, ACA1_374400, and ACA1_112650) during excystment. Among these four genes, one encodes for nicotinamide N-methyltransferase (NNMT; ACA1_112650), and the three others for hypothetical proteins. As NNMT is involved in the regulation of methylation potential, affecting DNA and histone epigenetic modifications, the authors hypothesized that this enzyme could regulate the expression of critical genes during excystment [86].

The degradation of cellulose is a critical step in the breakdown of the cyst wall during excystment and the importance of cellulases, enzymes that degrade cellulose, during this cellular process has been discussed by several works [81,87,88] indicating their pivotal role during the emergence of trophozoites from cysts. Indeed, the role of cellulases during excystment was further established by the finding that the treatment of *A. castellanii* cysts by exogenous cellulases at 7.5 U/mL for 24 h can stimulate the emergence of trophozoites [10]. Nevertheless, a cysticidal effect was observed on *A. castellanii* with high doses of cellulase from 500 U/mL to 1500 U/mL [89]. Furthermore, cyst treatment with proteases, such as pepsin and collagenase, also stimulated trophozoite emergence from cysts, but to a lower extent compared to cellulase at the same enzyme concentration [10]. Moreover, trophozoites excysted in the presence of cellulase displayed a three times shorter doubling time compared to the control in the absence of the enzyme, indicating that cyst wall degradation products generated by cellulase promote trophozoite proliferation. Moreover, no effect was observed on excystment stimulation with chitinase treatment [10], confirming the controversy for the presence, or the low accessibility, of chitin in the *Acanthamoeba* cyst wall [49,50,80,90]. The main cellular steps and endogenous proteins involved in both encystment and excystment processes are presented in Figure 4.

Although the precise composition of the operculum remains unclear due to isolation difficulties, it is hypothesized to contain cellulose organized differently from the rest of the cyst wall, making it a potential target for cellulases during excystment. Indeed, cellulose fibrils were previously shown to be organized in a layer-like manner and appeared unarranged and without orientation in the rest of the cyst wall [41]. The specific digestion of the operculum by cellulases during the excystment of *A. castellanii*, while the rest of the cyst wall remains intact, is still unresolved. One possibility is that the operculum may have a unique cellulose organization which would be more susceptible to cellulase activity than the rest of the cyst wall. Additionally, a specific localized activity of some cellulases, among the cellulase genes encoded in the *A. castellanii* genome, could be driven by a specific structural enzyme configuration, leading to exclusive operculum localization. Signaling mechanisms could also regulate cellulase-specific expression during encystment or excystment. The regulation of cellulase expression, both spatially and temporally, would likely direct the enzyme activity to the operculum, allowing trophozoite emergence through the ostiole and preserving, at least initially, the cyst wall integrity during the excystment process. Furthermore, the involvement of other enzymes working synergistically with cellulases, such as proteases [10], could weaken or modify the operculum, enabling the efficient emergence of trophozoites. Further research focusing on the detailed biochemical and molecular analyses of the operculum and the regulatory mechanisms governing cellulase activity would provide deeper insights into this fascinating biological process.

Although the detailed stages of excystment and key observations provided by Chambers and Thompson [85] contribute significantly to our knowledge of *A. castellanii* biology, future research should focus on elucidating the structural analysis of the operculum and the molecular mechanisms governing its removal and trophozoite emergence, besides cellulase activity, as well as the environmental factors influencing the timing and success of excystment.

## 5. Conclusions and Future Directions

Understanding the complex life cycle of *A. castellanii*, particularly the processes of encystment and excystment, is pivotal for addressing the challenges posed by this free-living amoeba. The ability of *A. castellanii* to alternate between trophozoite and cyst forms underlines its remarkable adaptability and survival mechanisms, which contributes significantly to its resistance to disinfection methods.

Encystment enables *A. castellanii* to endure harsh environmental conditions by forming a robust cyst wall primarily composed of cellulose and other polysaccharides. This complex structure not only protects the amoeba from desiccation, extreme temperatures, and chemical agents but also complicates disinfection efforts. Insights into the biochemical and molecular mechanisms of cyst formation, particularly the role of cellulose synthase and cellulases, will reveal essential genes for this cellular process.

Excystment is less well-characterized but equally critical. Research has identified key phases and structural features of excystment, with cellulases thought to play a crucial role in the breakdown of cellulose during this process. However, the precise mechanisms regulating this process, especially the selective digestion of the operculum in *Acanthamoeba* sp., remain an area of active investigation.

## Data Availability

Data and contained within the article.
